# Biocompatible and antibacterial soy protein isolate/quaternized chitosan composite sponges for acute upper gastrointestinal hemostasis

**DOI:** 10.1093/rb/rbab034

**Published:** 2021-06-30

**Authors:** Zijian Wang, MeiFang Ke, Liu He, Qi Dong, Xiao Liang, Jun Rao, Junjie Ai, Chuan Tian, Xinwei Han, Yanan Zhao

**Affiliations:** 1 Department of Interventional Radiology, The First Affiliated Hospital of Zhengzhou University, Zhengzhou 450052, China; 2 Department of Urology, Zhongnan Hospital of Wuhan University, Wuhan 430071, China; 3 Department of Biomedical Engineering and Hubei Province Key Laboratory of Allergy and Immune Related Disease, School of Basic Medical Sciences, Wuhan University, Wuhan 430071, China; 4 Department of Clinical Laboratory, Hubei Provincial Hospital of Traditional Chinese Medicine, Wuhan 430061, China

**Keywords:** quaternized chitosan, soy protein isolate, biocompatibility, antibacterial, hemostasis

## Abstract

Innovative biomedical applications have high requirements for biomedical materials. Herein, a series of biocompatible, antibacterial and hemostatic sponges were successfully fabricated for the treatment of acute upper gastrointestinal bleeding (AUGB). Quaternized chitosan (QC) and soy protein isolate (SPI) were chemically cross-linked to obtain porous SPI/QC sponges (named SQS-*n*, with *n* = 30, 40, 50 or 60 corresponding to the weight percentage of the QC content). The chemical composition, physical properties and biological activity of SQS-*n* were investigated. SQS-*n* could support the adhesion and proliferation of L929 cells while triggering no obvious blood toxicity. Meanwhile, SQS-*n* exhibited good broad-spectrum antibacterial activity against both gram-positive bacteria (*Staphylococcus aureus*) and gram-negative bacteria (*Escherichia coli*). The *in vivo* hemostatic effect of SQS-*n* was evaluated using three different bleeding models. The results revealed that SQS-50 performed best in reducing blood loss and hemostatic time. The overall hemostatic effect of SQS-50 was comparable to that of a commercial gelatin sponge. The enhanced antibacterial and hemostatic activities of SQS-*n* were mainly attributed to the QC component. In conclusion, this work developed a QC-functionalized hemostatic sponge that is highly desirable for innovative biomedical applications, such as AUGB.

## Introduction

Acute upper gastrointestinal bleeding (AUGB) refers to acute bleeding caused by diseases of the oesophagus, stomach, duodenum, pancreatic duct and bile duct above the flexor ligament [1, 2]. The amount of bleeding is expected to reach 1000 ml in critical cases, which leads to peripheral circulation failure in a short time [3]. In the clinic, patients with AUGB are conventionally given gastroscopy and antishock treatments, such as an intravenous injection of a balanced salt solution or normal saline (N.S.) [4, 5]. Gastroscopy can be used to easily inspect the bleeding sites, and a titanium clip can be used to stop bleeding [6, 7]. However, the non-degradable titanium clip may remain in the body for a long time and cause a series of discomforts. Biodegradable hemostatic materials, such as gelatin and chitosan sponges, are suitable to solve this problem [8–11]. The flexible mechanical arm of a gastroscope can precisely place hemostatic sponges at bleeding sites to achieve compression hemostasis. Subsequently, waste hemostatic sponges can be excreted or degraded by endogenous digestive enzymes without being removed. To date, gastroscopy-assisted compression hemostasis (GACH) is still immature, and few hemostatic materials have been customized for AUGB treatment.

The biological activities of hemostatic sponges need to be optimized according to the environment in which they are applied [12]. The upper gastrointestinal tract is not a sterile site, and a large number of microbial communities in the upper gastrointestinal tract are parasitic [13, 14]. Effected by food, digestive enzymes, pH values and other physiochemical factors, probiotics and pathogenic bacteria interact to maintain the steady state of the internal environment [15]. Many types of pathogenic bacteria may proliferate when AUGB occurs and then lead to secondary infection. On the other hand, hemostatic sponges absorb a large amount of blood components, increase pathogenic bacteria proliferation, and eventually aggravate local infection. Thus, good antibacterial activity of hemostatic sponges is required for special applications, such as GACH. In the USA, the biosafety of medical devices is strictly supervised by the Food and Drug Administration [16]. Currently, biocompatibility evaluations play an important role in the preclinical research stage of hemostatic sponges [17, 18].

Natural polymers, such as soy protein isolate (SPI), chitosan (CS), gelatin and alginate, are biocompatible, biodegradable and hypoimmunogenic [19]. Applications of these natural polymers have been extended to many biomedical fields, including hemostatic sponges [20–22]. SPI is the richest plant protein worldwide [23, 24]. Our group has reported that SPI-based biomaterials are hemostatic in a rabbit liver hemorrhaging model [25]. SPI has good hemostatic activity and biocompatibility but poor machinability. As previously reported, this drawback of SPI can be solved by chemical modification using other macromolecules, such as chitosan and gelatin [26]. Chitosan is the random copolymer of d-glucosamine and *N*-acetyl-d-glucosamine units [27]. The positively charged CS molecules deprotonate to form insoluble precipitates when reacting with an alkaline SPI solution [28]. Thus, it is difficult to prepare SPI/CS mixtures for subsequent chemical cross-linking reactions. Quaternized chitosan (QC) is a water-soluble chitosan derivative with enhanced antibacterial activity [29]. In this work, QC is an ideal substitute for chitosan.

**Figure 1.**
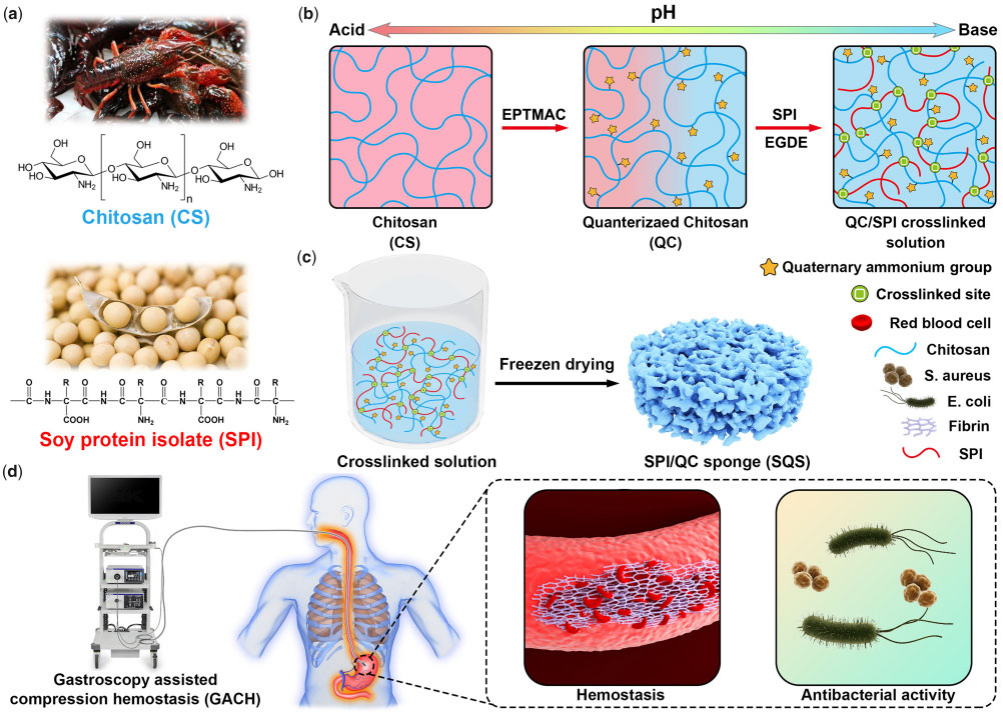
Schematic illustration of the components, preparation and applications of biocompatible, antibacterial and hemostatic soy protein isolate/QC sponges. (**a**) Chitosan (CS) and SPI were obtained from crustaceans and soybean, respectively; (**b**) CS was chemically modified with EPTMAC to obtain QC and then cross-linked with SPI. (**c**) The obtained cross-linked solution was freeze dried to obtain a series of SPI/QC composite sponges (SQS-*n*). (**d**) SQS-*n* was capable of killing bacteria and stopping bleeding, which are highly desirable for the treatment of AUGB

Inspired by the excellent biosafety and bioactivity of natural macromolecules, our aim was to fabricate a series of all biomass-derived hemostatic sponges using the freeze-drying technique. A schematic illustration of this work is shown in Fig. 1. The raw materials, including SPI and chitosan, were extracted from soybean and crustaceans (Fig. 1a). Then, water-soluble QC was synthesized from chitosan, physically blended with an alkaline SPI solution, and chemically cross-linked with ethylene glycol diglycidyl ether (EGDE) (Fig. 1b). The cross-linked solutions were freeze dried to fabricate a series of porous SPI/QC sponges, which were termed SQS-*n* (Fig. 1c). It is presumed that SQS-*n* has good biocompatibility, broad-spectrum antibacterial activity and enhanced hemostatic effects, which are highly desirable for innovative biomedical applications, such as AUGB (Fig. 1d). Herein, the effects of the QC content on the structural, physicochemical and biological properties of SQS-*n* were investigated in detail. Evaluations of the hemostatic effect of SQS-*n* were performed using three different bleeding models.

## Materials and methods

### Materials

QC (Ds > 95%) was purchased from YIHAOTIAN Biotechnology Co., Ltd. (Jiangsu, China). Soy protein isolate (SPI, Mw: 2.05 × 10^5^) was obtained from DuPont Protein Technology Co., Ltd. (Hubei, China). Mouse lung fibroblast cells (L929) were kindly supplied by Shanghai Institutes for Biological Science, Chinese Academy of Science (Shanghai, China). Dulbecco's modified Eagle’s medium (DMEM, high glucose), fetal bovine serum, 0.25% trypsin, penicillin, ampicillin, streptomycin and phosphate-buffered saline (PBS) were purchased from Thermo Fisher Scientific Co., Ltd. (Waltham, MA, USA). The MTT kit was obtained from DOJINDO Co., Ltd. (Shanghai, China). The live/dead bacteria staining kit was purchased from YEASEN Biotechnology Co., Ltd. (Shanghai, China). EGDE, dimethyl sulfoxide (DMSO) and NaOH were purchased from Sinopharm Chemical Reagent Co., Ltd. (Shanghai, China). All other reagents were of analytical purity.

### Fabrication of SPI/QC composite sponges

SPI/QC composite sponges were fabricated in one step using a freeze-drying technique. In accordance with previous literature [30], a 10 wt% SPI solution was sequentially prepared by dispersing 10 g SPI powder into 60 g distilled water (DW) and 30 g alkaline 5 wt% NaOH solution. A 6 wt% QC solution was prepared by dissolving 6 g QC powder into 94 g alkaline 1.5 wt% NaOH solution. QC and SPI solutions were mechanically blended to prepare a series of homogeneous SPI/QC mixtures with different QC contents (W_QC_, the weight percentage of QC) of 30, 40, 50 and 60%. Then, EGDE (20% total weight of QC and SPI raw materials) was added to the QC/SPI mixtures to form a chemical cross-linked network [31]. After stirring for 30 min, these mixtures were centrifuged at 3000 rpm for 15 min to remove air bubbles, poured into a customized mold and dried using a vacuum freeze dryer (FD-1A, BIOCOOL, Beijing). Then, the obtained products were washed with DW to remove the residual solvents and freeze dried again. In this work, these SPI/QC sponges were named SQS-*n* (*n* = 30, 40, 50 and 60), where the first S refers to SPI, Q refers to QC and the second S refers to sponge.

### Physiochemical characterizations

Optical images of SQS-*n* were captured using a digital camera (iPhone 11, Apple, USA) and its macrostructure was visualized by micro-CT scanning (IP224, Bruker, Germany). The outward and cross-sectional morphology of SQS-*n* (*n* = 30, 40, 50, 60) was observed using scanning electron microscopy (SEM) (VEGA3, TESCAN, Czech). Fourier transform infrared spectroscopy (FT-IR) (TNZ1-5700, Nicolet, USA) was performed at wavenumbers ranging from 4000 cm^−1–500^ cm^−1^. Wide angle X-ray diffraction (D-Advance, Bruker, USA) was carried out at diffraction angles ranging from 5° to 80°. The compressive properties of the SQS-*n* were analyzed using a universal testing machine (Instron 3340, ITW, USA). The swelling ratio (SR) of SQS-*n* was measured as reported previously [32].

### *In vitro* degradation test

SQS-*n* (*n* = 30, 40, 50 and 60) was cut into 1 × 1 × 0.5 cm^3^ pieces and then weighed using an analytical balance. SQS-*n* was incubated with simulated gastric juice (SGJ) at 37°C with constant stirring. The formula of SGJ was as follows: 2.0 g sodium chloride, 3.2 g pepsin and 7.0 ml hydrochloric acid, with DW added to a volume of 1000 ml. After 8 h of incubation, the samples were rinsed with DW, freeze dried and weighed again. The degradation rate (DR) was calculated using the following formula:

$$\mathrm{IR}\left(\%\right)=[1-\frac{\mathrm{NS}-\mathrm{NP}}{\mathrm{NB}-\mathrm{NP}}]\times 100,$$

where W0 and W1 refer to the average weight before and after degradation, respectively.

### *In vitro* antibacterial tests

*Escherichia coli* (*E. coli*) and *Staphylococcus aureus* (*S. aureus*) were used to evaluate the antibacterial activity of SQS-*n*. *Escherichia coli* and *S. aureus* are aerobic bacteria and were cultured in accordance with general protocols [33]. Briefly, 10 µl stored bacterial fluid was seeded onto Luria-Bertani (LB) solid medium using a plate streaking method, followed by incubation at 37°C overnight. A single bacterial clone was transferred into 5 ml LB liquid medium and amplified at 37°C with constant stirring overnight. The obtained proliferative bacterial fluid was centrifuged at 3000 rpm for 10 min to remove the medium and resuspended in 10 ml 0.85 wt% N.S. for further study. The concentration of the bacterial suspension was measured using ultraviolet-visible spectrophotometry and then adjusted to 1 × 10^8^ CFU/ml.

Clone formation assay [34]: SQS-*n* was cut into 1 × 1 × 0.5 cm^3^ pieces and then incubated with 2 ml *E. coli* or *S. aureus* bacterial suspension at 37°C with constant stirring for 5 h. The treated bacterial suspension was stepwise diluted with 0.85% N.S. and homogeneously seeded onto LB plates. The untreated bacterial suspension served as a blank control, and the ampicillin-treated bacterial suspension served as a positive control. After incubation overnight, the bacterial clones were visible to the naked eye. Optical images of the LB plates were captured using a digital camera (iPhone 11, Apple), and the number of bacterial clones was counted manually. The inhibition rate (IR) was calculated using the following formula:

$$\mathrm{IR}\left(\%\right)=\left[1-\frac{\mathrm{NS}-\mathrm{NP}}{\mathrm{NB}-\mathrm{NP}}\right]\times 100,$$

where NS, NP and NB refer to the average clone number in the sample group, positive control group and blank control group, respectively.

Bacterial proliferation assay [35]: the 100 µl treated bacterial suspension obtained in section 2.4.1 was added to 10 ml LB liquid medium. Then, the samples were transferred into a 37°C thermostatic oscillator for bacterial proliferation. At each time interval, 200 µl bacterial liquid was added to a 96-well tissue culture plate. The optical density (OD) value at a wavelength of 600 nm was detected using a microplate reader (MULTISCAN FC, Thermo, USA). As a result, proliferation curves of the treated bacteria were drawn.

Bacterial adhesion assay [36]: the SQS-*n* samples obtained in section 2.4.1 were dehydrated in an ascending series of ethanol and then vacuum-dried to remove residual solvent. The morphology of bacteria that adhered to the surface of SQS-*n* was observed using SEM (VEGA3, TESCAN, Czech). Before SEM observation, all the samples were coated with a gold layer to render them electrically conductive.

Live/dead bacterial staining assay [12]: the survival of bacteria was assessed by the live/dead bacterial staining method. A commercial kit was used in this study. Live bacteria were stained with DMAO (green), and dead bacteria were stained with EthD-III (red). A 1-ml treated bacterial suspension was added to a 1.5-ml tube, and then, 1 µl DMAO and 2 µl EthD-III dyes were added. After incubation in the dark for 15 min, 10 µl of the bacterial suspension was dropped onto a glass slide. A laser confocal microscope (LCS-SP8-STED, Leica, Germany) was used to capture images of live and dead bacteria.

### Cytocompatibility evaluations

MTT assay: the cytotoxicity of SQS-*n* (*n* = 30, 40, 50, 60) toward L929 cells was evaluated using the MTT assay. Extracts of SQS-*n* were prepared by incubating SQS-*n* powder with an appropriate amount of DMEM for 72 h. L929 cells were seeded in three 96-well tissue culture plates at a density of 1500 cells/well. After incubation for 24 h, the culture medium was replaced with the extracts. Culture medium without the extracts served as the negative control, culture medium without the extracts and L929 cells served as the blank control. After incubation for 1–3 days, 20 μl MTT solution was added to each well and incubated for 4 h. The medium was then replaced with 150 μl DMSO and slightly shaken for 10 min. The OD value at a wavelength of 490 nm was detected using a microplate reader (MULTISCAN FC, Thermo).

Cell adhesion assay: SQS-*n* (*n* = 30, 40, 50, 60) was cut into 0.8 × 0.8 × 0.2 cm^3^ pieces and placed on the bottom of 24-well tissue culture plates. L929 cells were seeded on the upper surface of SQS-*n* cells at a density of 5 × 10^4^ cells/well. After incubation for 48 h, the samples were fixed with a 4% paraformaldehyde (PFA) solution for 30 min and then dehydrated in an ascending series of ethanol. The morphology of L929 cells that adhered to the surface of SQS-*n* was observed using SEM (VEGA3, TESCAN, Czech).

### Hemocompatibility evaluation

The hemocompatibility evaluations were approved by the Animal Care & Welfare Committee of Wuhan University and carried out in accordance with the ‘Guidelines and Regulations for the use and care of Animals of the Review Board of Hubei Medical Laboratory Animal Center’. Fresh whole blood was obtained from a healthy New Zealand rabbit and then placed in a heparinized anticoagulative tube. The blood samples were diluted with 0.9% N.S. at a ratio of 1:1.25 for further study.

Hemolysis testing: SQS-*n* (*n* = 30, 40, 50, 60) was washed with 0.9% N.S. three times. The samples were incubated with 10 ml N.S. at 37°C for 30 min. Then, 0.2 ml diluted blood was added to each sample group, positive control group (DW) and negative control group (N.S.). After incubation for 60 min, all the samples were centrifuged, and optical images were captured using a digital camera (iPhone 11, Apple). The OD value of the supernatant at a wavelength of 545 nm was detected using a microplate reader (MULTISCAN FC, Thermo). The hemolysis ratio (HR) was calculated using the following formula:

HR(%)=[(AS-AN)/(AP-AN)]×100,

where AS, AP and AN refer to the OD values of the sample group, positive control group and negative control group, respectively.

Blood contact testing: SQS-*n* was cut into 0.8 × 0.8 × 0.2 cm^3^ pieces and then incubated with 1 ml diluted whole blood for 1 h at 37°C. Then, the samples were fixed with a 4% PFA solution for 30 min and washed with PBS three times to remove unattached red blood cells. After dehydration in an ascending series of ethanol, the surface morphology of SQS-*n* was observed using SEM (VEGA3, TESCAN, Czech).

### *In vivo* hemostatic evaluations

This study was approved by the Animal Care & Welfare Committee of Wuhan University and carried out in accordance with the ‘Guidelines and Regulations for the use and care of Animals of the Review Board of Hubei Medical Laboratory Animal Center’. Twenty-four Sprague-Dawley (SD) rats and 12 New Zealand rabbits were obtained from the Experimental Animal Center of Three Gorges University. Before the operation, these animals were anesthetized by 3% (v/v) isoflurane inhalation.

Hemostatic test of the rat tail amputation model: the rat tail amputation model was prepared in accordance with a previous report [37]. Twelve SD rats were randomly divided into four groups, and the tail of each rat was cut into half its length. Based on the results of previous tests, SQS-50 is preferred for *in vivo* hemostatic tests. The bleeding tail was covered with a piece of SQS-50 with a diameter of 10 mm and height of 4 mm. After the tail bleeding stopped, the blood loss and hemostatic time data were recorded. For the negative control, tail bleeding was not treated. For the positive control, tail bleeding was treated with gauze and a commercial gelatin hemostatic sponge. At least three independent samples were applied for statistical analysis.

Hemostatic test in a rat/rabbit liver injury model: the hemostatic activity of SQS-50 was further evaluated using a rat/rabbit liver injury model. Briefly, 12 SD rats or New Zealand rabbits were randomly divided into four groups: untreated, medical gauze, gelatin sponge and SQS-50. All animals were fixed on a surgical corkboard, the abdomen of these animals was cut to remove the liver, and a small amount of abdominal dropsy was removed using a piece of gauze. A 10 mm surgical wound was made in the liver using a scalpel, and then, the wound was covered with a piece of SQS-50 with a diameter of 10 mm and height of 4 mm. The bleeding time and blood loss data were recorded. Wounds covered by medical gauze or a commercial gelatin sponge were set as positive controls. Wounds without treatment were used as negative controls. At least three independent samples were used for statistical analysis.

### Statistical analysis

All the statistical data were analyzed using SPSS 19.0 software. Quantitative data are expressed as the mean ± standard deviation. Statistical analysis was performed using one-way analysis of variance, and a *P*-values <0.05 was considered to indicate a significant difference.

## Results and discussion

### Preparation and characterization of SQS-*n*

In this work, a series of EGDE cross-linked SPI/QC composite sponges (SQS-*n*) were prepared using a freeze-drying technique. The preparation parameters for SQS-*n* were optimized according to our previous work [30]. The obtained SQS-*n* samples were further characterized by a variety of physicochemical and biological tests.

**Figure 2.**
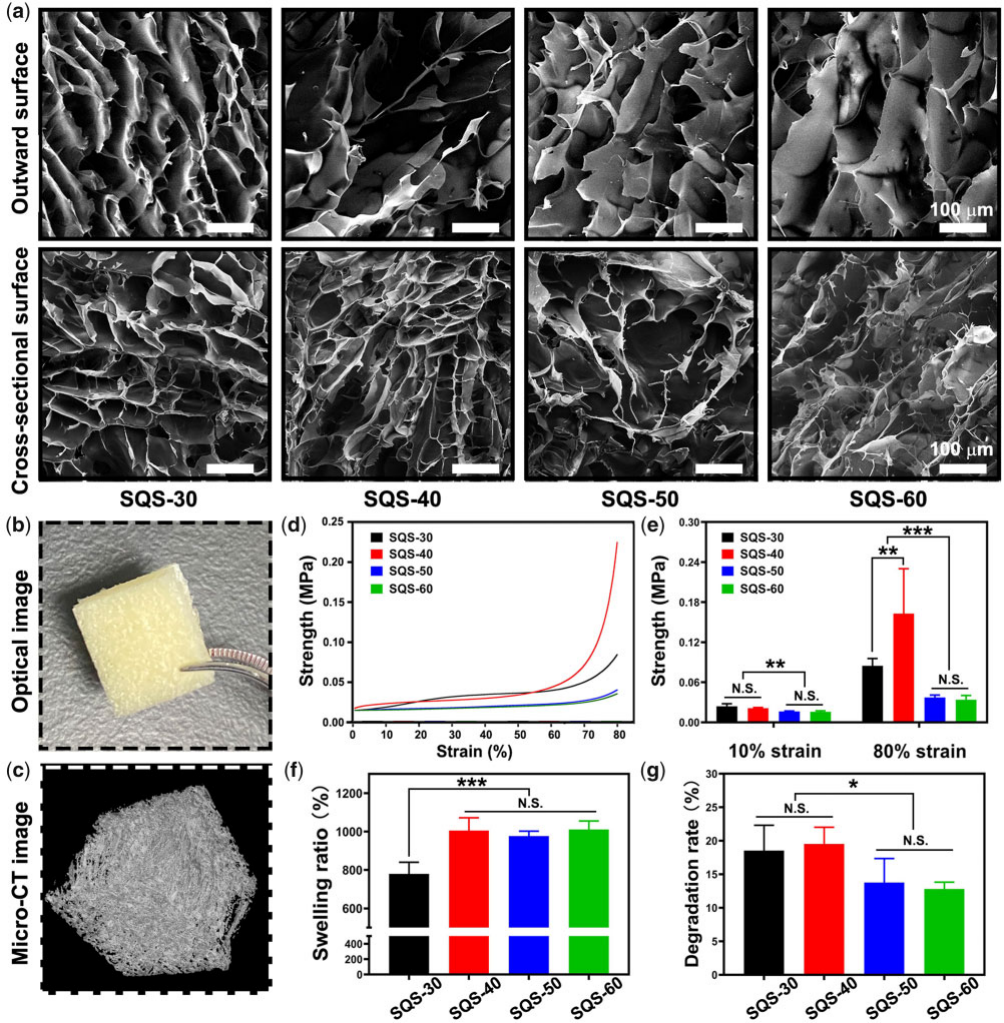
(**a**) Morphology of the upper surface and cross-section of SQS-*n*, scale bar = 100 µm; (**b**) optical images of SQS-50; (**c**) micro-CT images of SQS-50; (**d**) compressive curves of SQS-*n*; (**e**) results of the compressive strength at strains of 10% and 80%, **P < 0.01, ***P < 0.001; (**f**) results of the SR of SQS-*n*, ***P < 0.001; and (**g**) results of the *in vitro* DR of SQS-*n*, *P < 0.05

The outward surface and cross-sectional morphology of SQS-*n* was observed using SEM. As shown in Fig. 2a, all the SQS-*n* samples exhibited the typical porous microstructure. The roughness and pore size of the SQS-*n* samples increased gradually with increasing QC content. The porosity of SQS-*n* could facilitate the absorption of plasma in a short amount of time, activate red blood cells and coagulation factors, and thus enhance blood clotting.

Micro-CT observation was performed to visualize the macrostructure of SQS-*n*. In this work, SQS-50 was chosen as a representative of SQS-*n* (*n* = 30, 40, 50 and 60). SQS-50 was cut into pieces and exhibited a sponge-like appearance (Fig. 2b). The outward surface of SQS-50 was uniform without obvious cracks. The reconstructed micro-CT image is shown in Fig. 2c. Compared with that observed in the optical image, the porous structure of SQS-50 was clearer in the CT image.

A compression test was carried out to evaluate the mechanical properties of SQS-*n*. Compressive curves and compressive strength were chosen as the key indicators of mechanical properties [38]. As shown in Fig. 2d and e, the compression strength of SQS-*n* increased with increasing compressive strain from 0% to 80%. At 80% compressive strain, the compression strength was 0.085 ± 0.001 MPa for SQS-30, 0.163 ± 0.067 MPa for SQS-40, 0.037 ± 0.003 MPa for SQS-50 and 0.034 ± 0.006 MPa for SQS-60, respectively. The mechanical strength of SQS-*n* was determined by the rigid SPI framework as well as the cross-linked network between SPI, QC and EGDE. At 80% compressive strain, the compression strength of SQS-40 peaked, which could be attributed to the maximum cross-linking degree.

Porous sponges are capable of absorbing a large amount of plasma when applied as hemostatic materials. In this study, PBS was applied as a substitute for plasma to evaluate the SR of SQS-*n*. As shown in Fig. 2f, the SR was 780 ± 60% for SQS-30, 1005 ± 67% for SQS-40, 976 ± 26% for SQS-50 and 1011 ± 45% for SQS-60. A significant difference was observed between SQS-30 and the other groups (*P* < 0.001). The high absorption ability was mainly attributed to the porous spongy structure of the SQS-*n* samples.

The stomach is full of gastric juice. When a hemostatic sponge is used for GACH, it is decomposed by digestive enzymes. Hemostatic sponges should be stable enough to resist digestive enzymes. In this work, an *in vitro* degradation test was performed. Clinically, the gastric emptying time was <8 h. To imitate the physiological environment of patients, SQS-*n* was incubated with SGJ at 37°C with constant stirring for 8 h. As shown in Fig. 2g, the DRs were 18.5 ± 3.8% SQS-30, 19.5 ± 2.5% SQS-40, 13.8 ± 3.6% SQS-50 and 12.8 ± 1.0% SQS-60. The DR of SQS-*n* decreased significantly with increasing QC content, partly because gastric juice can digest proteins but not polysaccharides. Notably, the DR of SQS-*n* was relatively slow in the first 8 h, and SQS-*n* only worked after a few minutes in the early stage of hemostasis. Thus, we infer that the stability of SQS-*n* is sufficient for *in vivo* application.

### Chemical component of SQS-*n*

**Figure 3.**
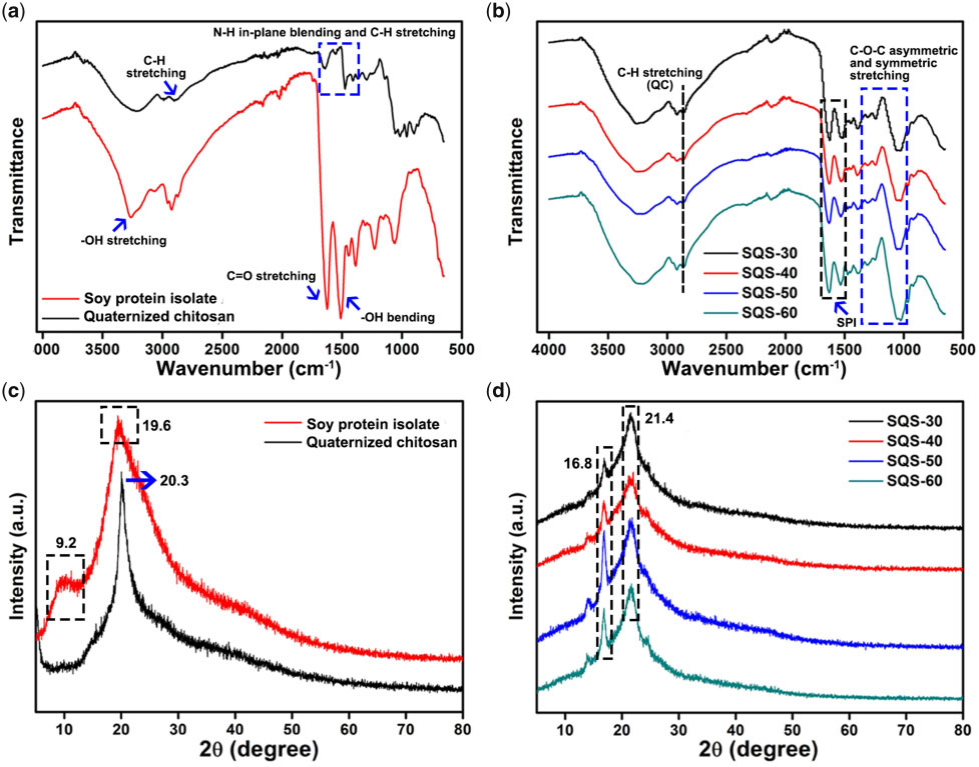
(**a**) FT-IR spectra of the raw materials, including SPI and QC; (**b**) FT-IR spectra of SQS-*n* (*n* = 30, 40, 50, 60); (**c**) XRD spectra of SPI and QC; and (**d**) XRD spectra of SQS-*n*

FT-IR was performed to investigate the chemical cross-linking of SQS-*n*. FT-IR spectra of the raw materials are shown in Fig. 3a. QC exhibited three characteristic peaks, including C-H stretching (2899 cm^−1^), C-H stretching (1639 and 1472 cm^−1^) of the methyl group and N-H in-plane blending (1560 cm^−1^) of the primary amine group on quaternary ammonium groups. SPI also showed three characteristic peaks at 3259, 1627 and 1510 cm^−1^, which were assigned to -OH stretching, C=O stretching and -OH bending, respectively. Figure 3b shows the FT-IR spectra of SQS-*n* (*n* = 30, 40, 50 and 60). The peak at 2899 cm^−1^ was assigned to the C-H stretching of QC, and the peak at 1628 cm^−1^ was assigned to the C=O stretching of SPI. Notably, the peak of -OH bending at 1510 cm^−1^ in the SPI group shifted slightly to 1512 cm^−1^ in the SQS-30 group, 1519 cm^−1^ in the SQS-40 group, 1529 cm^−1^ in the SQS-50 group and 1535 cm^−1^ in the SQS-60 group. Meanwhile, a series of C-O-C asymmetric and symmetric stretching peaks (1250–1020 cm^−1^) appeared among the SQS-*n* samples. These results confirmed that SPI and QC were cross-linked by EGDE.

The chemical composition of SQS-*n* was further identified by XRD spectra. As shown in Fig. 3c, SPI exhibited two characteristic peaks at 9.2° and 19.6°. The characteristic peak of QC was located at 20.3°. The XRD spectra of SQS-*n* are shown in Fig. 3d. We found that the characteristic peak at 9.2° disappeared, and the peaks at 19.6° and 20.3° shifted obviously to 21.4°. Several new peaks were found. In particular, the peak at 16.8° was marked, which might be attributed to the crystal structure of the QC/SPI cross-linked complex. These results were consistent with those of the FT-IR analysis.

### *In vitro* antibacterial evaluations

The upper gastrointestinal tract connects the stomach with the external environment. A large number of bacteria, including probiotics and pathogenic bacteria, are found in the stomach and oesophagus [39]. As the balance of the flora is broken by bleeding, pathogenic bacteria may proliferate, leading to a secondary infection at the hemorrhagic spot. An ideal hemostatic material for treating upper gastrointestinal bleeding should meet the high requirement of broad-spectrum antibacterial activity.

**Figure 4.**
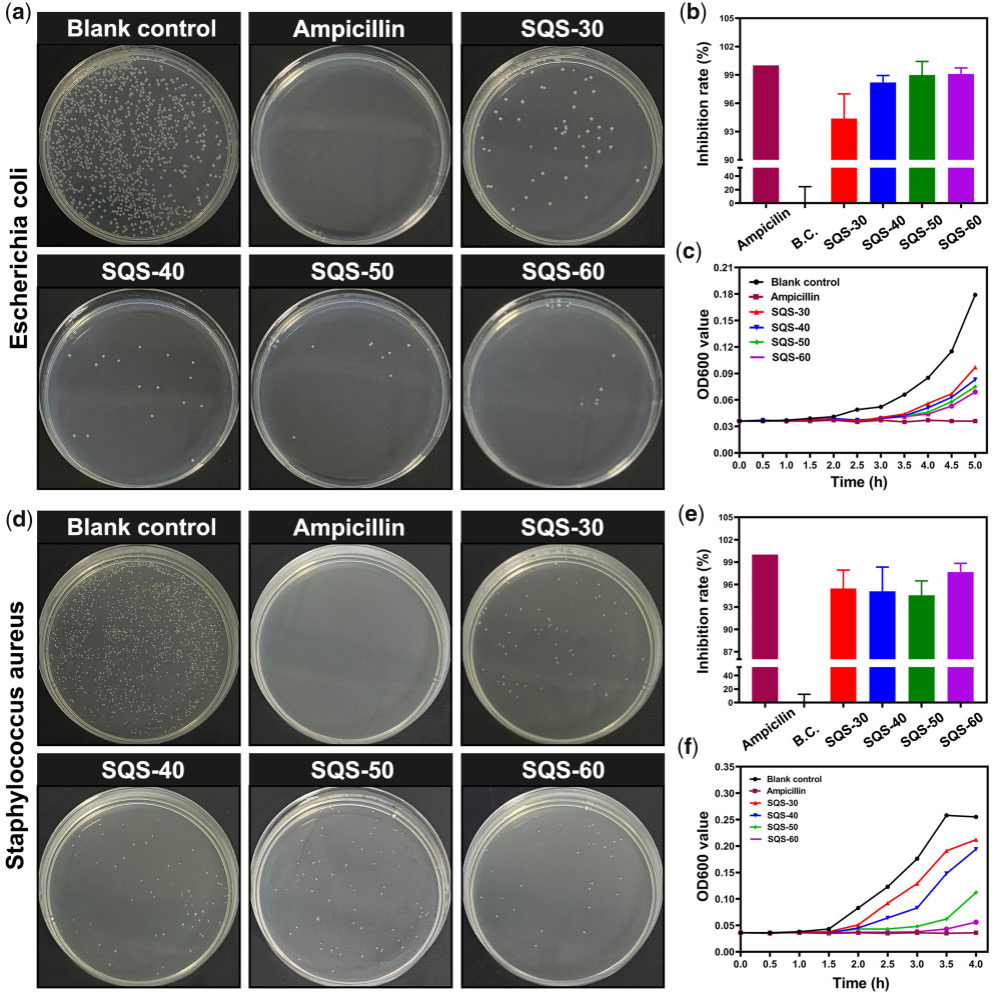
(**a**) Photographs of *E. coli* clones treated with SQS-*n* (*n* = 30, 40, 50, 60); ampicillin was used as the positive control; (**b**) statistical results of the number of *E. coli* clones; (**c**) proliferation curves of *E. coli*; (**d**) photographs of *S. aureus* clones; (**e**) statistical results of the number of *S. aureus* clones; and (**f**) proliferation curves of *S. aureus*

The bacterial suspension was incubated with SQS-*n* (*n* = 30, 40, 50, 60) for 5 h. For the clone formation assay, the treated bacterial suspension was homogeneously seeded on LB plates, and then, the number of bacterial clones was counted. Representative images of *E. coli* clones are shown in Fig. 4a. Compared with the blank control group, no *E. coli* clone formed in the ampicillin-treated group. The *E. coli* bacterial strain used in this study was sensitive to antibiotics (ampicillin). The bacterial clones in the SQS-30, SQS-40, SQS-50 and SQS-60 groups were obviously less abundant than those in the blank control group. The quantitative analysis results of the bacterial clone are expressed as the IR and are shown in Fig. 4b. The IR of the blank control group was set as 0%, and that of the ampicillin-treated group was set as 100%. The IR was 94.4 ± 2.6% for the SQS-30 group, 98.2 ± 0.7% for the SQS-40 group, 98.9 ± 1.4% for the SQS-50 group and 99.1 ± 0.6% for the SQS-60 group. The overall antibacterial activity of the SQS-*n* samples increased with increasing the *n* value from 30 to 60. The viability of the treated *E. coli* was further evaluated by a bacterial proliferation assay. As shown in Fig. 4c, the value of OD600 increased gradually from 0 to 5 h, which was correlated with the bacterial density in the LB medium. After treatment with SQS-*n*, the proliferation rate of *E. coli* decreased obviously. In conclusion, the SQS-*n* samples exhibited good antibacterial activity against *E. coli*.

As a model organism of gram-positive bacteria, *S. aureus* was also used for *in vitro* antibacterial evaluations. Representative images of *S. aureus* clones are shown in Fig. 4d, and the quantitative results of the IR are shown in Fig. 4e. The IR was 95.5 ± 2.5% for the SQS-30 group, 95.1 ± 3.2% for the SQS-40 group, 94.6 ± 1.9% for the SQS-50 group and 97.7 ± 1.2% for the SQS-60 group. As shown in Fig. 4f, the value of OD600 decreased obviously after SQS-*n* treatment. These results suggested that the SQS-*n* samples were also antibacterial against *S. aureus*.

**Figure 5.**
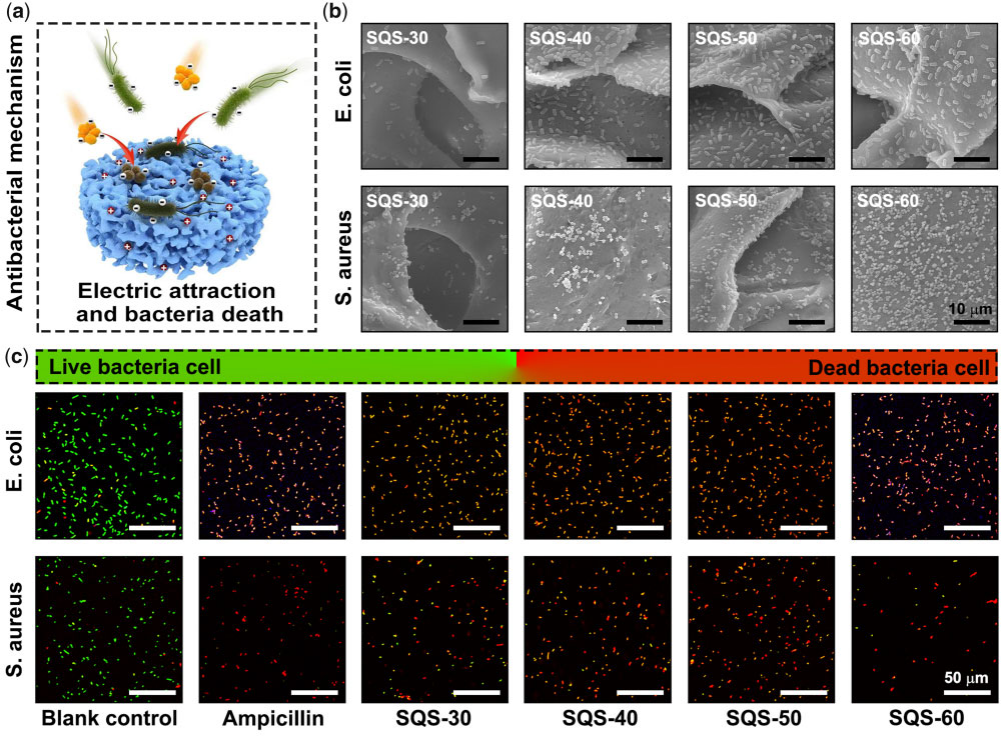
(**a**) Schematic diagrams of the *in situ* antibacterial mechanism of SQS-*n*; (**b**) morphology of *E. coli* and *S. aureus* adhering to the surface of SQS-*n* (*n* = 30, 40, 50, 60), scale bar = 10 µm; and (**c**) fluorescence images of live/dead bacteria staining of *E. coli* and *S. aureus*, scale bar = 50 µm

The antibacterial activity of the SQS-*n* samples can be attributed to the QC component, whose mechanism has been reported. As shown in Fig. 5a, the QC component of the SQS-*n* samples possesses a homogeneous cationic charge and can be electrostatically attracted with negatively charged bacteria. The amphiphilic molecular chain of QC can insert into the bacterial membrane, resulting in bacterial lysis and death. To verify the attraction between the oppositely charged QC and bacteria, the SQS-*n* samples were incubated with bacterial suspensions. The obtained SQS-*n* samples that adhered to bacteria were then dehydrated for SEM observation. As shown in Fig. 5b, *E. coli* and *S. aureus* were evenly distributed on the outer surface of the SQS-*n* samples. As the *n* value increased from 30 to 60, the bacterial density increased gradually, suggesting that the positive charge increased.

The antibacterial mechanism of QC is to destroy the bacterial membrane. However, not all bacteria with damaged membranes die immediately. In Fig. 4, we found that a few treated bacteria in wounds survived. To explain this phenomenon, a live/dead bacteria staining assay with greater sensitivity was performed. Live bacteria were stained with green DMAO dye, and dead bacteria were stained with red EthD-III dye. Any bacteria with a damaged membrane were dyed red. Representative fluorescence images are shown in Fig. 5c. Almost all the bacteria in the SQS-30, SQS-40, SQS-50 and SQS-60 groups were stained red, suggesting that the bacterial membrane was damaged by the QC molecular chain. Meanwhile, a small number of bacteria were dyed red and green at the same time and showed an orange color. These bacteria were injured but not completely killed. Some of these damaged bacteria could survive and form new bacterial communities.

### Biocompatibility of SQS-*n*

**Figure 6.**
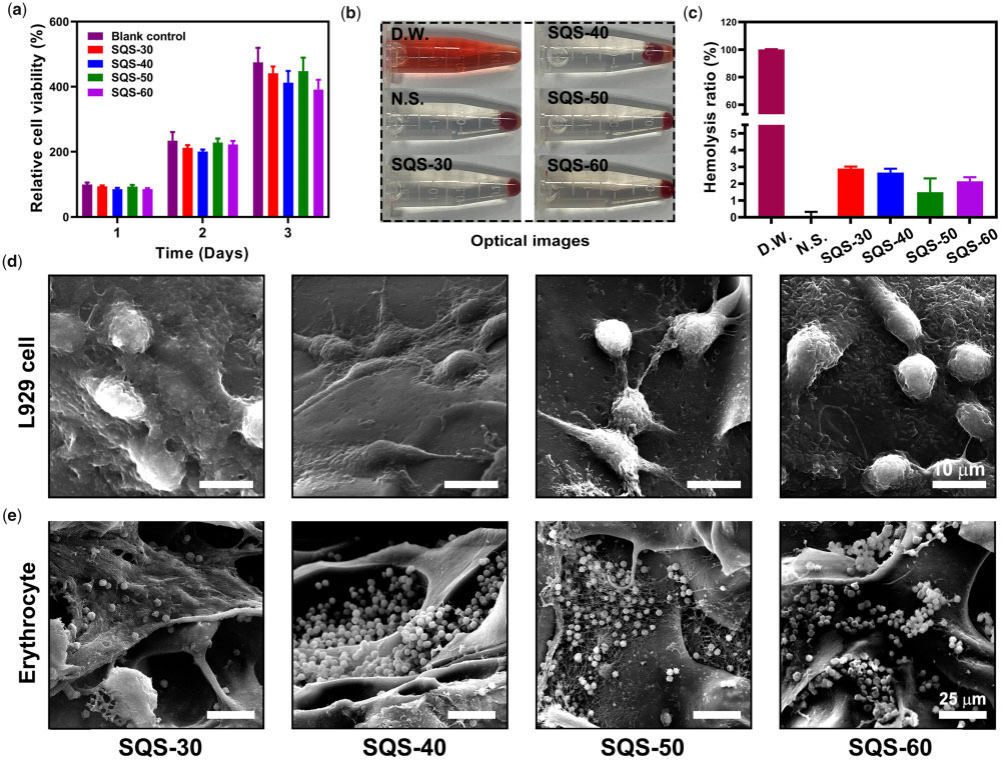
(**a**) Proliferation of L929 cells treated with the extracts of SQS-*n* (*n* = 30, 40, 50, 60); (**b** and **c**) optical images of hemolytic red blood cells, and the statistical results of the hemolysis ratio; (**d**) SEM images of L929 cells seeded onto the upper surface of SQS-*n*, scale bar =10 µm. (**e**) SEM images of erythrocytes adhering to SQS-*n*, scale bar = 25 µm

As an ideal hemostatic material, SQS-*n* sponges should possess good biocompatibility, such as cytocompatibility and hemocompatibility. Herein, an MTT assay was performed to evaluate the cytocompatibility of the SQS-*n* samples. Mouse lung fibroblast cells (L929) were cultured with extracts of the SQS-*n* samples. As shown in Fig. 6a, the viability of L929 cells in the blank control group was set as 100% on day 1, 234 ± 27% on day 2 and 475 ± 441% on day 3. This result indicated that the L929 cells were healthy and proliferative. After treatment with the SQS-*n* samples, the relative viability of L929 cells decreased slightly. Notably, the viability of L929 cells decreased along with the increase in QC content, suggesting that QC was potentially toxic [36]. Usually, cell viability from the MTT assay is required to be no <80%. Thus, the SQS-30, SQS-40 and SQS-50 samples were eligible.

Hemolysis tests were carried out to investigate the hemocompatibility of SQS-*n*. Representative images of the hemolytic red blood cells are shown in Fig. 6b, and the quantitative results of the hemolysis ratio (HR) are shown in Fig. 6c. In this study, the HR in DW group was set as 100%, and that in N.S. group was set as 0%. The HRs were 2.89 ± 0.12% for the SQS-30 group, 2.66 ± 0.23% for the SQS-40 group, 1.49 ± 0.83% for the SQS-50 group and 2.14 ± 0.24% for the SQS-60 group. The hemolysis ratio of the SQS-*n* samples met the high requirements of international norms (HR < 5%).

To further verify the biocompatibility of the SQS-*n* samples, a cell contact test was carried out. First, L929 cells were seeded on the upper surface of SQS-*n* (*n* = 30, 40, 50 and 60). As shown in Fig. 6d, the SQS-*n* samples could support the adhesion of L929 cells. The cell adhesion ability of biomaterials could be determined by a series of factors, such as the surface roughness, mechanical strength, cytotoxicity and hydrophobic properties [40]. L929 cells spread best on SQS-40 and SQS-50. Erythrocytes were also seeded onto SQS-*n*. As shown in Fig. 6e, a large number of erythrocytes adhered to the SQS-30, SQS-40, SQS-50 and SQS-60 samples, partly owing to the electrostatic interactions between the positively charged QC and negatively charged erythrocytes. The SQS-*n* samples exhibited non-toxicity or minimal toxicity to erythrocytes. Notably, the erythrocytes in the SQS-50 sample were coated with a large amount of fibrin, suggesting that the SQS-50 sample initiated the coagulation pathway in blood.

### *In vivo* hemostatic performance of SQS-*n*

The purpose of this work was to develop a novel hemostatic sponge for the treatment of AUGB. The unique anatomical structure and function of the gastrointestinal tract lead to high requirements for hemostatic materials. In this work, we prepared a series of SPI/QC composite sponges (SQS-*n*). The incorporation of the QC component was supposed to functionalize SQS-*n* with enhanced antibacterial activity and hemostatic effects.

In this part, we evaluated the hemostatic effect of SQS-*n* samples in a series of animal models, including a rat tail amputation model, rat liver injury model and rabbit liver injury model. Medical gauze and commercial gelatin sponges both served as positive controls. Based on the biocompatibility and antibacterial activity results, the SQS-50 sample was preferred among the SQS-*n* samples (*n* = 30, 40, 50, 60) and was used as the experimental group. For each animal model, hemostatic time and blood loss were chosen as the key indicators of hemostatic effects.

**Figure 7.**
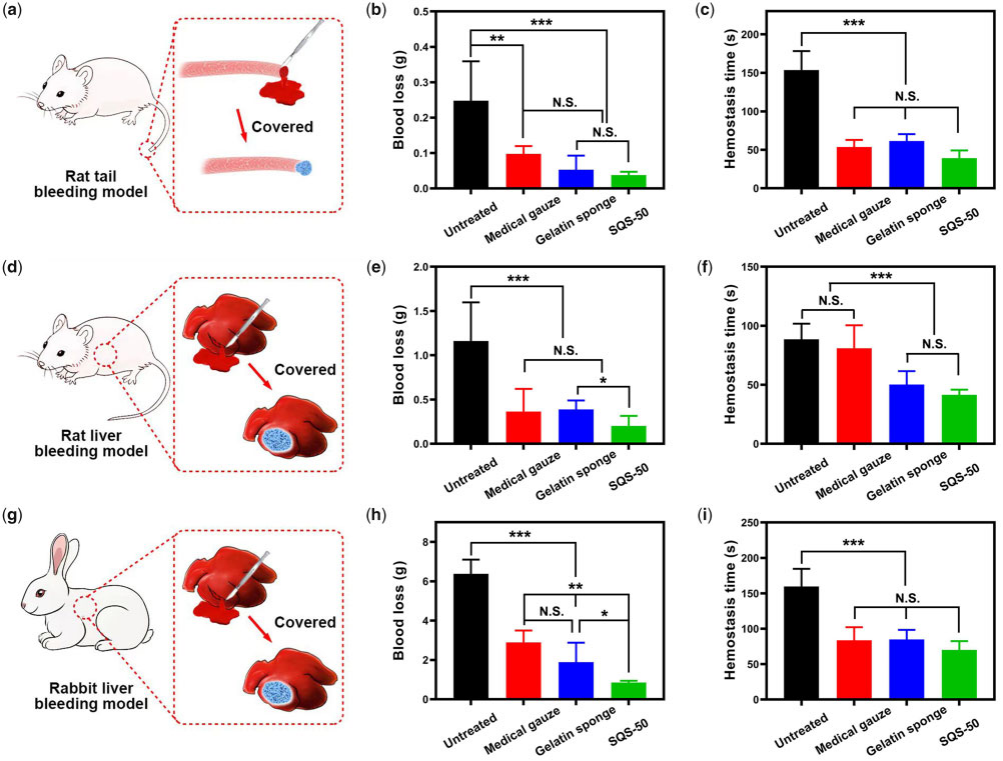
(**a**) Diagram of the preparation of the rat tail amputation model; (**b** and **c**) quantitative results of the blood loss and hemostasis time in a rat tail amputation model. Medical gauze and a commercial gelatin sponge were set as the positive controls, **P < 0.05, ***P < 0.01; (**d**) Diagram of the preparation of the rat liver bleeding model; (**e** and **f**) quantitative results of the blood loss and hemostasis time in a rat liver bleeding model, *P < 0.05, ***P < 0.001. (**g**) Diagram of the preparation of the rabbit liver bleeding model and (**h** and **i**) quantitative results of the blood loss and hemostasis time in a rabbit liver bleeding model, *P < 0.05, **P < 0.01, ***P < 0.001

Diagrams of the rat tail amputation model are shown in Fig. 7a. The tail of each rat was cut into half its length and then covered with a piece of SQS-50, medical gauze or gelatin sponge. The quantitative results of the blood loss and hemostasis time are shown in Fig. 7b and c. Compared with those in the untreated group, the blood loss and hemostatic time in the medical gauze, gelatin sponge and SQS-50 groups were all decreased significantly (*P* < 0.01). The blood loss was 0.248 ± 0.112 g in the untreated group, 0.097 ± 0.022 g in the medical gauze group, 0.053 ± 0.041 g in the gelatin sponge group and 0.038 ± 0.010 g in the SQS-50 group. No significant difference was observed between the SQS-50 and gelatin sponge groups, indicating that they had equivalent hemostatic effects.

The animal liver is rich in blood, and it bleeds when it is damaged. The liver injury model has been widely used for hemostasis evaluation. Diagrams of the rat/rabbit liver injury model are shown in Fig. 7d and g. The abdomen of all animals was cut to remove the liver. A 10-mm surgical wound was created and then treated with hemostatic materials. The quantitative results of the rat liver injury model are shown in Fig. 7e and f, and the quantitative results of the rabbit liver injury model are shown in Fig. 7h and i. The SQS-50 sample performed best, especially in reducing blood loss.

The hemostatic mechanism of the SQS-50 sample remains to be determined. We believe that mechanical compression plays a major role in stopping bleeding. The SQS-50 sponge can provide external pressure to the bleeding spot, seal the broken ends of blood vessels, and thereby reduce blood loss. Meanwhile, the coagulation pathways activated by the SQS-50 sample are also important. The spongy structure of the SQS-50 sample can quickly absorb a large amount of plasma, condense red blood cells and coagulation factors and promote blood clotting. The surface roughness, net charge and hydrophobic properties of SQS-50 can also participate in the activation of coagulation pathways.

In conclusion, our results confirm that the SQS-50 sample is biocompatible, antibacterial and hemostatic. The excellent properties of SQS-50 are highly desirable for the treatment of AUGB. Because of technical and ethical issues, we failed to fabricate an *in situ* animal model of AUGB. Thus, the strength of the conclusions of this study is reduced. We plan to cooperate with clinical researchers to supplement bedside research in follow-up work.

## Conclusions

In summary, we successfully fabricated a series of EGDE cross-linked SPI/QC composite sponges (SQS-*n*) via a freeze-drying method. The physical properties of the SQS-*n* samples, such as the compressive strength and SR, could be optimized by adjusting the QC content. The SQS-*n* samples exhibited minimal toxicity toward L929 cells and erythrocytes. Notably, the SQS-*n* samples were proven to have broad-spectrum antibacterial activity against both gram-positive bacteria (*S. aureus*) and gram-negative bacteria (*E. coli*). The antibacterial mechanism of SQS-*n* could be attributed to the positive charge and amphiphilic molecular chain of QC. *In vivo* hemostasis tests further demonstrated that SQS-50 could reduce blood loss and hemostasis time in three different bleeding models. The hemostatic effect of the SQS-50 sample was comparable to that of a commercial gelatin sponge. The products produced in this work were biocompatible, antibacterial and hemostatic, and they could be applied for the bedside treatment of AUGB.

## Funding

The authors thank Prof. Yun Chen and Prof. Xinghuan Wang from Wuhan University for their great support. This work was supported by the Medical Science Advancement Program (Clinical Medicine) of Wuhan University (TFLC2018003), the Horizontal Research Program of Zhengzhou University (24110005), the Science and Technology Department of Hubei Province Key Project (2018ACA159) and the Chinese Central Special Fund for Local Science and Technology Development of Hubei Province (2018ZYYD023).

*Conflict of interest statement*. None declared.
